# Comparison of clinician diagnosis of COVID-19 with real time polymerase chain reaction in an adult-representative population in Sweden

**DOI:** 10.1186/s12931-023-02315-7

**Published:** 2023-01-11

**Authors:** Eman Quraishi, Chiamaka Jibuaku, Daniil Lisik, Göran Wennergren, Jan Lötvall, Fredrik Nyberg, Linda Ekerljung, Madeleine Rådinger, Hannu Kankaanranta, Bright I. Nwaru

**Affiliations:** 1 Krefting Research Centre, Institute of Medicine, University of Gothenburg, Gothenburg, Sweden; 2 Department of Paediatrics, Sahlgrenska Academy at University of Gothenburg, Gothenburg, Sweden; 3 School of Public Health and Community Medicine, Institute of Medicine, University of Gothenburg, Gothenburg, Sweden; 4 Faculty of Medicine and Health Technology, Tampere University, Tampere, Finland; 5 Department of Respiratory Medicine, Seinäjoki Central Hospital, Tampere, Finland; 6 Wallenberg Centre for Molecular and Translational Medicine, University of Gothenburg, Gothenburg, Sweden

**Keywords:** COVID-19, RT-PCR, Clinical diagnosis, ICD-10, Sensitivity, Specificity, Positive predictive value, Negative predictive value, Youden index, Asthma, COPD, Validation

## Abstract

**Background:**

Due to the high transmissibility of SARS-CoV-2, accurate diagnosis is essential for effective infection control, but the gold standard, real-time reverse transcriptase-polymerase chain reaction (RT-PCR), is costly, slow, and test capacity has at times been insufficient. We compared the accuracy of clinician diagnosis of COVID-19 against RT-PCR in a general adult population.

**Methods:**

COVID-19 diagnosis data by 30th September 2021 for participants in an ongoing population-based cohort study of adults in Western Sweden were retrieved from registers, based on positive RT-PCR and clinician diagnosis using recommended ICD-10 codes. We calculated accuracy measures of clinician diagnosis using RT-PCR as reference for all subjects and stratified by age, gender, BMI, and comorbidity collected pre-COVID-19.

**Results:**

Of 42,621 subjects, 3,936 (9.2%) and 5705 (13.4%) had had COVID-19 identified by RT-PCR and clinician diagnosis, respectively. Sensitivity and specificity of clinician diagnosis against RT-PCR were 78% (95%CI 77–80%) and 93% (95%CI 93–93%), respectively. Positive predictive value (PPV) was 54% (95%CI 53–55%), while negative predictive value (NPV) was 98% (95%CI 98–98%) and Youden’s index 71% (95%CI 70–72%). These estimates were similar between men and women, across age groups, BMI categories, and between patients with and without asthma. However, while specificity, NPV, and Youden’s index were similar between patients with and without chronic obstructive pulmonary disease (COPD), sensitivity was slightly higher in patients with (84% [95%CI 74–90%]) than those without (78% [95%CI 77–79%]) COPD.

**Conclusions:**

The accuracy of clinician diagnosis for COVID-19 is adequate, regardless of gender, age, BMI, and asthma, and thus can be used for screening purposes to supplement RT-PCR.

**Supplementary Information:**

The online version contains supplementary material available at 10.1186/s12931-023-02315-7.

## Introduction

As COVID-19 continues to spread in waves across the world, rapid and accurate diagnosis are essential tools to identify, isolate, and appropriately manage patients, thereby decreasing the rate of infectivity, morbidity, and mortality [1]. Robust and rapid diagnosis of COVID-19 also aids in surveillance, management and control of disease, epidemiologic characterization, contact tracing, and decision making for public health purposes [2, 3]. However, at the beginning of the pandemic, diagnosis was challenging, primarily because of the disparate symptoms manifested by those infected, ranging from mild or no symptoms to life-threatening presentations [4]. In response, various diagnostic approaches were employed, which are classified based on their underlying indications and principles.

Diagnostic approaches currently being used for COVID-19 can be broadly divided into two basic categories: clinical and in vitro diagnostics [5–7]. The clinical diagnostic methods are based on assessment of symptoms, imaging techniques, and laboratory tests. Findings from these methods can be non-specific and insufficient to provide compelling evidence of COVID-19 infection [7]. The diagnostic methods are commonly divided into: (a) nucleic acid-based assays, in which RNA of the virus causing COVID-19, severe acute respiratory syndrome coronavirus 2 (SARS-CoV-2), is amplified; and (b) serological assays, in which antibodies/antigens specific to SARS-CoV-2 are targeted [8, 9]. Real-time reverse transcriptase-polymerase chain reaction (RT-PCR) is one of the most sensitive and widely implemented nucleic acid-based assays [10, 11], commonly considered to be the “gold standard” for diagnosis of COVID-19 [12, 13].

Not all COVID-19 patients or suspected cases end up getting an RT-PCR test, but rather are examined by a clinician and thus get classified using the recommended International Classification of Disease (ICD) codes for COVID-19. It is unclear to what extent these clinician-based diagnoses correctly identify true COVID-19 cases. While only a few studies have evaluated the accuracy of COVID-19 diagnosis, majority of these have usually compared topography with RT-PCR. To our knowledge, the accuracy of the recommended ICD codes has not yet been validated in a population-based setting. Doing so will help to ascertain as to what extent they can be used independently or complimentarily to RT-PCR for diagnosing COVID-19. The aim of this study was to compare primary and secondary care diagnosis of COVID-19 by a clinician using the recommended ICD codes with RT-PCR in an adult-representative population. Furthermore, in comparing the two diagnostic approaches, we evaluated whether accuracy of diagnosis differed by age, gender, BMI, and pre-COVID-19 obstructive airway diseases and comorbidities.

## Methods

**Fig. 1:**
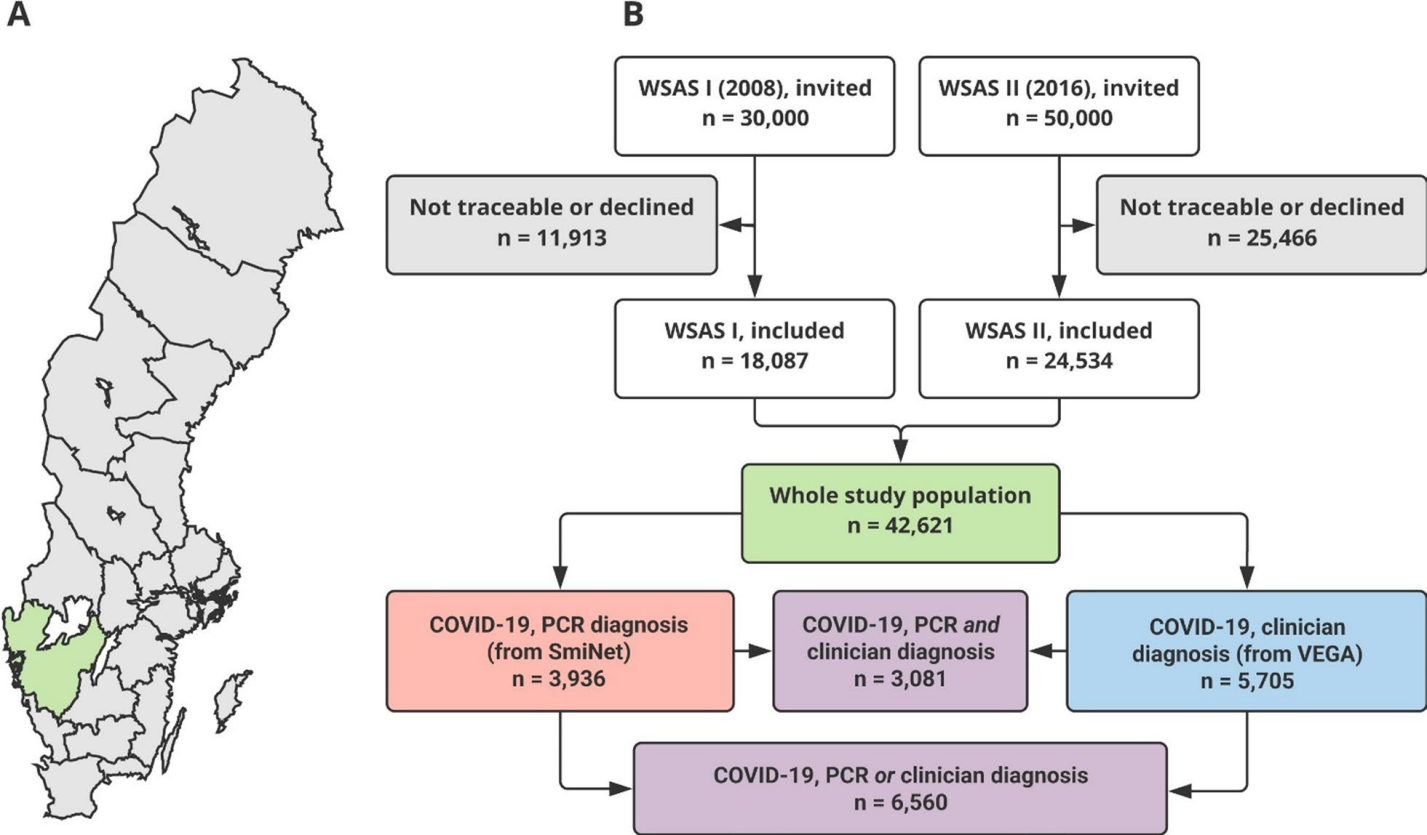
Flowchart of study cohort. **A** Map of Sweden. Marked in green is Västra Götaland county, from which the participants of this cohort (West Sweden Asthma Study [WSAS]) were recruited. B Fflowchart of the recruitment process to WSAS I and II, respectively, and the subsets of the final cohort (marked in green) that got COVID-19 diagnosed either through real-time reverse transcriptase-polymerase chain reaction (RT-PCR; marked in red), by a clinician (marked in blue), or both/either (marked in purple). SmiNet: national monitoring system with a register of RT-PCR-diagnosed COVID-19 cases. VEGA: regional (Västra Götaland) database with primary and secondary care data

This analysis was based on the ongoing West Sweden Asthma Study (WSAS), which is a large population-representative longitudinal cohort study of adults (16–75 years at enrolment) randomly recruited from Västra Götaland county in western Sweden. WSAS constitutes of 42,621 subjects, of which 18,087 were recruited in 2008, while 24,534 were recruited in 2016. A flowchart of the study cohort is shown in Fig. 1. All participants had pre-COVID questionnaire data collected in 2008 and/or 2016, covering various demographic, environmental, and socio-demographic data, as well as the presence of obstructive airway diseases/symptoms and comorbidities.

Using the unique personal identification number given to all residents in Sweden, we collected information on COVID-19 diagnosis for all participants in WSAS, both from the register hosting clinician diagnosis in Västra Götaland region (VEGA) and that hosting RT-PCR diagnosis (SmiNet). While VEGA is a regional database system by Region Västra Götaland that collects information on primary and secondary care contacts for western Sweden, SmiNet is a national database for reporting RT-PCR COVID-19 diagnosis run by the Swedish Public Health Agency. From both registers, we collected data on COVID-19 diagnosis up until 30th September 2021. In VEGA, COVID-19 cases were identified based on the recommended ICD-10 codes, including U07.1, U07.2, U08.9, U09.9, and U10.9. Estimates of the accuracy of clinician diagnosis were determined and compared against RT-PCR as the reference standard. The study was approved by the regional ethics board at the University of Gothenburg as well as the national ethics board.

### Statistical analysis

All statistical analyses were carried out using Stata/SE version 17.0 (StataCorp, College Station, Texas, USA). The distribution of pre-COVID-19 demographics by COVID-19 diagnosis and by patterns of COVID-19 diagnosis using clinician assessment and RT-PCR were compared using the Pearson Chi-square test. To assess the performance of clinician diagnosis of COVID-19 against RT-PCR, we calculated sensitivity, specificity, negative predictive value (NPV), and positive predictive value (PPV), each with its respective 95% confidence interval (95% CI) using Wilson’s method without continuity correction [14]. Youden’s index (sensitivity + specificity − 1) with 95% CI was obtained using the method based on the empirical proportion estimate proposed by Shan [15]. The Youden’s index as a measure of diagnostic accuracy measures the ability of a diagnostic test to balance between the sensitivity and specificity of the diagnostic test. Usually, a value of 50% is used as a cut-off for having an acceptable test result that meet empirical benchmark for diagnostic test to be administered for diagnostic purposes. Accuracy estimates were calculated for the entire study population as well as by age, gender, BMI, and pre-COVID-19 obstructive airway diseases and comorbidities. A significance level of 0.05 was used.

## Results

### Cohort characteristics

From the total of 42,621 subjects who participated in WSAS pre-COVID-19 questionnaires, 6560 COVID-19 cases were identified. Of these, 3936 were diagnosed with RT-PCR, 5705 were diagnosed by a clinician, and 3081 had a COVID-19 infection diagnosed using both methods (Fig. 1). A comparison of pre-COVID-19 demographic factors between COVID-19 cases and non-cases is presented in Table 1. COVID-19 cases were younger than non-cases, but they were comparable regarding gender, smoking habits, BMI, growing up on a farm, and rural residence during childhood. Although COVID-19 cases were slightly more educated than non-cases, the two groups did not differ regarding social class classification. Regarding pre-COVID presence of respiratory diseases, COVID-19 cases and non-cases did not differ in reported clinician-diagnosed chronic obstructive pulmonary disease (COPD), but COVID-19 cases were slightly more likely to report clinician-diagnosed asthma, particularly allergic asthma, than non-cases. COVID-19 cases were also slightly more likely to report any respiratory symptom in the last year than non-cases. Regarding the presence and number of comorbidities, there were no differences between COVID-19 cases and non-cases (Table 1).

**Table 1:** Distribution of pre-COVID demographics by COVID-19 infection

	All	COVID-19 cases	Non-cases	*P*-value
	*N* = 42,621	*N* = 6,560	*N* = 36,061	
	*n* (%)	*n* (%)	*n* (%)	
*Age, years*				
≤ 30	2,760 (6.48)	589 (8.98)	2,171 (6.02)	< 0.001
31–44	8,855 (20.78)	1,795 (27.36)	7,060 (19.58)	
45–60	12,167 (28.55)	2,491 (37.97)	9,676 (26.83)	
> 60	18,839 (44.20)	1,685 (25.69)	17,154 (47.57)	
*Gender*				
Female	23,227 (54.50)	3,735 (56.94)	19,492 (54.05)	< 0.001
Male	19,394 (45.50)	2,825 (43.06)	16,569 (45.95)	
*Smoking*				
Non-smoker	26,131 (61.31)	4,268 (65.06)	21,863 (60.63)	< 0.001
Ex-smoker	10,171 (23.86)	1,425 (21.72)	8,746 (24.25)	
Smoker (or stopped < 1year ago)	6,319 (14.83)	867 (13.22)	5,452 (15.12)	
*Body mass index (BMI), kg/m*^*2*^				
< 25	21,099 (49.50)	3,219 (49.07)	17,880 (49.58)	0.734
25–29.99…	15,682 (36.79)	2,430 (37.04)	13,252 (36.75)	
≥ 30	5,840 (13.70)	911 (13.89)	4,929 (13.67)	
Raised on a farm				
No	37,976 (89.10)	5,932 (90.43)	32,044 (88.86)	< 0.001
Yes	4,645 (10.90)	628 (9.57)	4,017 (11.14)	
*Rural residence during childhood*				
No	29,808 (69.94)	4,711 (71.81)	25,097 (69.6)	< 0.001
Yes	12,813 (30.06)	1,849 (28.19)	10,964 (30.4)	
*Highest education attained*				
Less than high school	6,824 (16.01)	730 (11.13)	6,094 (16.9)	< 0.001
High school	19,355 (45.41)	3,144 (47.93)	16,211 (44.95)	
Tertiary education	16,442 (38.58)	2,686 (40.95)	13,756 (38.15)	
*Social class classification†*				
1 (managers)	2,276 (5.34)	368 (5.61)	1,908 (5.29)	< 0.001
2 (professionals)	8,011 (18.80)	1,354 (20.64)	6,657 (18.46)	
3 (technicians and associate professionals)	5,645 (13.24)	906 (13.81)	4,739 (13.14)	
4 (office support workers)	3,440 (8.07)	478 (7.29)	3,012 (8.35)	
5 (service and sale workers)	9,508 (22.31)	1,557 (23.73)	7,875 (21.84)	
6 (skilled agriculture/forestry/fishery workers)	1,440 (3.38)	211 (3.22)	1,255 (3.48)	
7 (craft and related trade workers)	2,540 (5.96)	366 (5.58)	2,174 (6.03)	
8 (plant and machine operators/assemblers)	1,907 (4.47)	277 (4.22)	1,630 (4.52)	
9 (elementary occupations)	7,854 (18.43)	1,043 (15.9)	6,811 (18.89)	
0 (military personnel)	0 yy	0 (0)	0 (0)	
*Clinician-diagnosed COPD*				
No	41,374 (97.07)	6,401 (97.58)	34,973 (96.98)	0.009
Yes	1,247 (2.93)	159 (2.42)	1,088 (3.02)	
*Clinician-diagnosed asthma*				
No	38,965 (91.42)	5,884 (89.7)	33,081 (91.74)	< 0.001
Yes	3,656 (8.58)	676 (10.3)	2,980 (8.26)	
*Allergic asthma (asthma + rhinitis)*				
No	39,489 (92.65)	5,941 (90.56)	33,548 (93.03)	< 0.001
Yes	3,132 (7.35)	619 (9.44)	2,513 (6.97)	
*Any respiratory symptom‡*				
No	30,357 (71.23)	4,520 (68.9)	25,837 (71.65)	< 0.001
Yes	12,264 (28.77)	2,040 (31.1)	10,224 (28.35)	
*Number of comorbidities§*				
0	16,952 (39.77)	2,602 (39.66)	14,350 (39.79)	0.911
1	12,433 (29.17)	1,906 (29.05)	10,527 (29.19)	
≥ 2	13,236 (31.06)	2,052 (31.28)	11,184 (31.01)	
*Number of "severe" comorbidities (COPD, diabetes)*				
0				
1	39,262 (92.12)	6,121 (93.31)	33,141 (91.9)	0.001
2	3,164 (7.42)	413 (6.3)	2,751 (7.63)	
	195 (0.46)	26 (0.4)	169 (0.47)	
*Number of "moderately severe" comorbidities (asthma, hypertension)*				
0				
1	31,093 (72.95)	4,997 (76.17)	26,096 (72.37)	< 0.001
2	10,450 (24.52)	1,426 (21.74)	9,024 (25.02)	
	1,078 (2.53)	137 (2.09)	941 (2.61)	
*Number of "mild" comorbidities (eczema, rhinitis, sleep disorder)*				
0				
1	21,790 (51.13)	3,161 (48.19)	18,629 (51.66)	< 0.001
≥ 2	13,818 (32.42)	2,166 (33.02)	11,652 (32.31)	
	7,013 (16.45)	1,233 (18.80)	5,780 (16.03)	

^†^ Following the classification of Standard för svensk yrkesklassificering (SSYK), based on International Standard Classification of Occupation 2008 (ISCO-08)

^‡^ Within the last year, any of the following: (a) attack of shortness of breath, or waking up with chest tightness, or any wheeze, longstanding cough; (b) dyspnea walking on level ground at normal pace, or recurrent wheezing; or (c) productive cough for periods of ≥ 3 months

^§^ Asthma, COPD, diabetes, eczema, hypertension, rhinitis, sleep disorder

COPD: chronic obstructive pulmonary disease

### Relation of pre-COVID demographic factors to patterns of COVID-19 diagnosis

Table 2 presents the number of COVID-19 cases diagnosed by a clinician, RT-PCR, and different combinations of the two approaches by pre-COVID-19 demographic factors. Being diagnosed by a clinician, regardless of RT-PCR diagnosis, increased with increasing age and increasing number of comorbidities. It was also more common among those with less than high school education than those with higher educational levels, and slightly more common among overweight or obese subjects than those with BMI < 25 kg/m^2^. However, the proportion of clinician diagnosed COVID-19 was similar between males and females, between smokers and non-smokers, and by social class classification levels (Table 2).

**Table 2:** Distribution of pre-COVID demographics by different patterns of COVID-19 diagnosis using clinical assessment and real-time reverse transcriptase-polymerase chain reaction (RT-PCR)

	Clinician diagnosis*		RT-PCR diagnosis		Clinician and RT-PCR diagnosis		Clinician diagnosis but no RT-PCR diagnosis		RT-PCR diagnosis but no clinician diagnosis	
	n = 5705		n = 3936		n = 3081		n = 2624		n = 855	
	n (%)	P-value	n (%)	P-value	n (%)	P-value	n (%)	P-value	n (%)	P-value
*Age, years*										
≤ 30	478 (81.15)	< 0.001	362 (61.46)	< 0.001	251 (42.61)	0.001	227 (38.54)	< 0.001	111 (18.85)	< 0.001
31–44	1,492 (83.12)		1,116 (62.17)		813 (45.29)		579 (37.83)		303 (16.88)	
45–60	2,188 (87.84)		1,548 (62.14)		1,245 (49.98)		943 (37.86)		303 (12.16)	
> 60	1,547 (91.81)		910 (54.01)		772 (45.28)		775 (45.99)		138 (8.19)	
Gender										
Female	2,449 (86.69)	0.563	1,784 (63.15)	< 0.001	1,408 (49.84)	< 0.001	1,041 (36.85)	< 0.001	376 (13.31)	0.563
Male	3,256 (87.18)		2,152 (57.62)		1,673 (44.79)		1,583 (42.38)		855 (12.82)	
Smoking										
Non-smoker	3,696 (86.60)	0.15	2,634 (61.72)	< 0.001	2,062 (48.31)	< 0.001	1,634 (38.28)	< 0.001	572 (13.40)	0.15
Ex-smoker	1,261 (88.49)		834 (58.53)		670 (47.02)		591 (41.47)		164 (11.51)	
Smoker (or stopped < 1year ago)	748 (86.27)		468 (53.98)		349 (40.25)		399 (46.02)		119 (13.73)	
Body mass index (BMI), kg/m2										
< 25	2,732 (84.87)	< 0.001	1,953 (60.67)	0.206	1,466 (45.54)	0.027	1,266 (39.33)	0.206	487 (15.13)	< 0.001
25–29.99…	2,163 (89.09)		1,460 (60.08)		1,194 (49.09)		970 (39.92)		267 (10.99)	
≥ 30	810 (88.91)		523 (57.41)		422 (46.32)		388 (42.59)		101 (11.09)	
*Raised on a farm*										
No	5,141 (86.67)	0.026	3,598 (60.65)	0.001	2,807 (47.32)	0.078	2,334 (39.35)	0.001	791 (13.33)	0.026
Yes	564 (89.81)		338 (53.82)		274 (43.63)		290 (46.18)		64 (10.19)	
Rural residence during childhood										
No	4,075 (86.50)	0.073	2,829 (60.05)	0.893	2,193 (46.55)	0.281	1,882 (39.95)	0.893	636 (13.50)	0.073
Yes	1,630 (88.16)		1,107 (59.87)		888 (48.03)		742 (40.31)		219 (11.84)	
*Highest education attained*										
Less than high school	669 (91.64)	< 0.001	396 (54.25)	0.003	335 (45.89)	0.809	334 (45.75)	0.003	61 (8.36)	< 0.001
High school	2,721 (86.55)		1,900 (60.43)		1,477 (46.98)		1,244 (39.57)		423 (13.45)	
Tertiary education	2,315 (86.19)		1,640 (61.06)		1,269 (47.24)		1,046 (38.94)		371 (13.81)	
*Social class classification†*										
1 (managers)	333 (90.49)	0.035	226 (61.41)	0.012	191 (51.90)	< 0.001	142 (38.59)	0.012	35 (9.51)	0.035
2 (professionals)	1,159 (85.60)		823 (60.78)		628 (46.38)		531 (39.22)		195 (14.40)	
3 (technicians and associate professionals)	791 (87.31)		588 (64.90)		473 (52.21)		318 (35.10)		115 (12.69)	
4 (office support workers)	425 (88.91)		278 (58.16)		225 (47.07)		200 (41.84)		53 (11.09)	
5 (service and sale workers)	1,346 (86.45)		917 (58.90)		706 (45.34)		630 (41.10)		211 (13.55)	
6 (skilled agriculture/forestry/fishery workers)	173 (81.99)		125 (59.24)		87 (41.23)		86 (40.76)		38 (18.01)	
7 (craft and related trade workers)	322 (87.98)		231 (63.11)		187 (51.09)		135 (36.89)		44 (12.02)	
8 (plant and machine operators/assemblers)	251 (90.61)		163 (58.84)		137 (49.46)		114 (41.16)		26 (9.39)	
9 (elementary occupations)	905 (86.77)		585 (56.09)		447 (42.86)		458 (43.91)		138 (13.23)	
0 (military personnel)	0 (0)		0 (0)		0 (0)		0 (0)		0 (0)	
*Clinician-diagnosed COPD*										
No	5,558 (86.83)	0.038	3,862 (60.33)	< 0.001	3,019 (47.16)	0.041	2,539 (39.67)	< 0.001	843 (13.17)	0.038
Yes	147 (92.45)		74 (46.54)		62 (38.99)		85 (53.46)		12 (7.55)	
*Clinician-diagnosed asthma*										
No	5,125 (87.10)	0.341	3,558 (60.47)	0.022	2,799 (47.57)	0.004	2,326 (39.53)	0.022	759 (12.90)	0.341
Yes	580 (85.80)		378 (55.92)		282 (41.72)		298 (44.08)		96 (14.20)	
*Allergic asthma (asthma + rhinitis)*										
No	5,168 (86.99)	0.868	3,586 (60.36)	0.065	2,813 (47.35)	0.054	2,355 (39.64)	0.065	773 (13.01)	0.868
Yes	537 (86.75)		350 (56.54)		268 (43.30)		269 (43.46)		82 (13.25)	
*Any respiratory symptom‡*										
No	3,912 (86.55)	0.135	2,801 (61.97)	< 0.001	2,193 (48.52)	< 0.001	1,719 (38.03)	< 0.001	608 (13.45)	0.135
Yes	1,793 (87.89)		1,135 (55.64)		888 (43.53)		905 (44.36)		247 (12.11)	
Number of comorbidities§										
0	2,224 (85.47)	0.006	1,639 (62.99)	< 0.001	1,261 (48.46)	0.027	963 (37.01)	< 0.001	378 (14.53)	0.006
1	1,662 (87.20)		1,149 (60.28)		905 (47.48)		757 (39.72)		244 (12.80)	
≥ 2	1,819 (88.65)		1,148 (55.95)		915 (44.59)		904 (44.05)		233 (11.35)	
Number of "severe" comorbidities (COPD, diabetes)										
0	5,307 (86.70)	0.018	3,706 (60.55)	0.002	2,892 (47.25)	0.235	2,415 (39.45)	0.002	814 (13.30)	0.018
1	377 (91.28)		214 (51.82)		178 (43.10)		199 (48.18)		36 (8.72)	
2	21 (80.77)		16 (61.54)		11 (42.31)		10 (38.46)		5 (19.23)	
Number of "moderately severe" comorbidities (asthma, hypertension)										
0	4,309 (86.23)	0.003	3,077 (61.58)	< 0.001	2,389 (47.81)	0.015	1,920 (38.42)	< 0.001	688 (13.77)	0.003
1	1,269 (88.99)		797 (55.89)		640 (44.88)		629 (44.11)		157 (11.01)	
2	127 (92.70)		62 (45.26)		52 (37.96)		75 (54.74)		10 (7.30)	
Number of "mild" comorbidities (eczema, rhinitis, sleep disorder)										
0	2,732 (86.43)	0.409	1,949 (61.66)	0.001	1,520 (48.09)	0.015	1,212 (38.34)	0.001	429 (13.57)	0.409
1	1,890 (87.26)		1,303 (60.16)		1,027 (47.41)		863 (39.84)		276 (12.74)	
≥ 2	1,083 (87.83)		684 (55.47)		534 (43.31)		549 (44.53)		150 (12.17)	

COPD: chronic obstructive pulmonary disease. RT-PRC: real-time reverse transcriptase-polymerase chain reaction

^*^ Records of any of the following ICD-10 codes: U07.1, U07.2, U08.9, U09.9, U10.9

^†^ Following the classification of Standard för svensk yrkesklassificering (SSYK), based on International Standard Classification of Occupation 2008 (ISCO-08)

^‡^ Within the last year, any of the following: (a) attack of shortness of breath, or waking up with chest tightness, or any wheeze, longstanding cough; (b) dyspnea walking on level ground at normal pace, or recurrent wheezing; (c) productive cough for periods of ≥ 3 months

^§^ Asthma, COPD, diabetes, eczema, hypertension, rhinitis, sleep disorder

Being diagnosed by RT-PCR, regardless of clinician diagnosis, was more common among females than males and among non-smokers than current or past smokers. On the other hand, RT-PCR diagnosis was less common among those who grew up on a farm than those who did not, among those who had less than high school education than those with higher education levels, among those with two or more comorbidities than those with one or none, among those aged over 60 years than younger subjects, and among those with COPD, asthma, or any respiratory symptom in the last year than those without these respiratory disorders. There was no significant difference in being diagnosed by RT-PCR by BMI, rural residence during childhood, or social class classification (Table 2).

Being diagnosed by both a clinician and by RT-PCR was more common in those aged 45–60 years than other age groups, among overweight subjects than those with BMI ≥ 30 kg/m^2^ or BMI < 25 kg/m^2^, and among females compared to males. Subjects with COPD, asthma, and those with any respiratory symptom in the last year were less likely to be diagnosed using this approach than those without these respiratory disorders. Similarly, fewer were diagnosed by both a clinician and by RT-PCR with increasing number of comorbidities, as well as among current smokers compared to non-smokers and past smokers. However, it did not differ by level of education, being raised on a farm, or rural residence during childhood (Table 2).

Being diagnosed by a clinician but not confirmed by RT-PCR was more common among those aged 60 years and above compared to the younger age groups. This was also more common in males than females, in current smokers than past smokers or non-smokers, in those who grew up on a farm than those who did not, in those who had less than high school education than those with higher education levels, and in those with COPD, asthma, or any respiratory symptom in the last year than those without these respiratory disorders. Additionally, being diagnosed by a clinician without RT-PCR confirmation increased with increasing number of comorbidities (Table 2). On the other hand, the proportion of subjects diagnosed by RT-PCR but not by a clinician decreased with increasing age. It was lower among those with COPD than those without, among those who grew up on a farm than those who did not, and it decreased with increasing number of comorbidities. In contrast, diagnosis by only RT-PCR was more common among those with higher than lower school education, and among those with BMI < 25 kg/m^2^ compared to overweight and obese subjects. For gender, asthma, and report of any respiratory symptom in the last year, there was no significant differences in being diagnosed by only RT-PCR (Table 2).

### Comparison of clinician diagnosis and RT-PCR diagnosis of COVID-19

In all subjects, of those diagnosed using RT-PCR, clinician diagnosis correctly identified 78% as positive, and of those ruled out as negative by RT-PCR, clinician diagnosis correctly ruled out 93%. The validation estimates are given as follows: sensitivity 0.78 (95% CI 0.77–0.80), specificity 0.93 (95% CI 0.93–93), PPV 0.54 (95% CI 0.53–0.55), NPV 0.98 (95% CI 0.98–0.98), and Youden’s index 0.71 (95% CI 0.70–0.72). These estimates did not differ between males and females, but the sensitivity increased with increasing age, ranging from 0.69 (95% CI 0.64–0.70) for those aged ≤ 30 years to 0.85 (95% CI 0.82–0.87) for those aged > 60 years (Table 3).

**Table 3:** Estimates and 95% confidence interval (95% CI) of sensitivity, specificity, positive predictive value, negative predictive value, and Youden’s index for COVID-19 clinical diagnosis against real-time reverse transcriptase-polymerase chain reaction (RT-PCR) in all COVID-19 cases, and by gender and age

	All COVID-19 cases *N* = 6,560 Estimate (95% CI)	Gender		Age groups			
		Males *n* = 19,394 Estimate (95% CI)	Females *n* = 23,227 Estimate (95% CI)	≤ 30 years *n* = 2,760 Estimate (95% CI)	31–44 years *n* = 8,855 Estimate (95% CI)	45–60 years *n* = 12,167 Estimate (95% CI)	> 60 years *n* = 18,839 Estimate (95% CI)
Sensitivity	0.78 (0.77–0.80)	0.79 (0.77–0.81)	0.78 (0.76–0.79)	0.69 (0.64–0.74)	0.73 (0.70–0.75)	0.80 (0.78–0.82)	0.85 (0.82–0.87)
Specificity	0.93 (0.93–0.93)	0.94 (0.94–0.94)	0.92 (0.92–0.93)	0.91 (0.89–0.92)	0.91 (0.91–0.92)	0.91 (0.91–0.92)	0.96 (0.95–0.96)
PPV	0.54 (0.53–0.55)	0.57 (0.56–0.59)	0.51 (0.50–0.53)	0.53 (0.48–0.57)	0.54 (0.52–0.57)	0.57 (0.55–0.59)	0.50 (0.47–0.52)
NPV	0.98 (0.98–0.98)	0.98 (0.98–0.98)	0.98 (0.97–0.98)	0.95 (0.94–0.96)	0.96 (0.95–0.96)	0.97 (0.97–0.97)	0.99 (0.99–0.99)
Youden’s index^1^	0.71 (0.70–0.72)	0.73 (0.71–0.75)	0.70 (0.68–0.72)	0.60 (0.55–0.65)	0.64 (0.61–0.67)	0.71 (0.69–0.73)	0.81 (0.79–0.83)

NPV: negative predictive value. PPV: positive predictive value

^1^ Sensitivity + specificity—1

Stratifying the results by BMI, sensitivity was lowest among those with BMI < 25 kg/m^2^ compared to those with higher BMI, but the specificity, PPV, and NPV were similar for all BMI groups (Table 4). While the sensitivity and PPV were higher among those without asthma, the specificity and NPV were similar between those with and without asthma. The specificity and NPV were also similar between those with and without COPD, but the sensitivity was higher among those with than those without COPD, in contrast to the case with asthma, while the PPV was higher among those without than among those with COPD (Table 4). Stratifying the results by the number of comorbidities, the specificity and NPV were similar across groups, but the sensitivity increased while the PPV decreased with increasing number of comorbidities (Table 4). With further division of comorbidities into “severe” (COPD, diabetes), “moderately severe” (asthma, hypertension), and “mild” conditions (eczema, rhinitis, and sleep disorders), different patterns of results were observed (Table 5). While the specificity was similar across groups, the sensitivity was highest and lowest in those with one and two “severe” comorbidities, respectively. For those with “moderately severe” comorbidities, sensitivity increased with increasing number of comorbidities, but the specificity remained similar across groups. For those with “mild” comorbidities, both the sensitivity and specificity were similar across groups (Table 5).

**Table 4:** Estimates and 95% confidence interval (95% CI) of sensitivity, specificity, positive predictive value, negative predictive value, and Youden’s index by pre-COVID BMI, comorbidity, and asthma/COPD for clinician diagnosis against real-time reverse transcriptase-polymerase chain reaction (RT-PCR) diagnosis of COVID-19

	Body mass index (BMI), kg/m^2^			Asthma		COPD		Number of comorbidities^1^		
	< 25 *n* = 21,099 Estimate (95% CI)	25–29.99… *n* = 15,682 Estimate (95% CI)	≥ 30 *n* = 5,840 Estimate (95% CI)	No *n* = 38,965 Estimate (95% CI)	Yes *n* = 3,656 Estimate (95% CI)	No *n* = 41,374 Estimate (95% CI)	Yes *n* = 1,247 Estimate (95% CI)	0 *n* = 16,952 Estimate (95% CI)	1 *n* = 12,433 Estimate (95% CI)	≥ 2 *n* = 13,236 Estimate (95% CI)
Sensitivity	0.75 (0.73–0.77)	0.82 (0.80–0.84)	0.81 (0.77–0.84)	0.79 (0.77–0.8)	0.75 (0.7–0.79)	0.78 (0.77–0.79)	0.84 (0.74–0.90)	0.77 (0.75–0.79)	0.79 (0.76–0.81)	0.8 (0.77–0.82)
Specificity	0.93 (0.93–0.94)	0.93 (0.93–0.94)	0.93 (0.92–0.93)	0.93 (0.93–0.94)	0.91 (0.9–0.92)	0.93 (0.93–0.93)	0.93 (0.91–0.94)	0.94 (0.93–0.94)	0.93 (0.93–0.94)	0.93 (0.92–0.93)
PPV	0.54 (0.52–0.56)	0.55 (0.53–0.57)	0.52 (0.49–0.56)	0.55 (0.53–0.56)	0.49 (0.45–0.53)	0.54 (0.53–0.56)	0.42 (0.34–0.5)	0.57 (0.55–0.59)	0.54 (0.52–0.57)	0.50 (0.48–0.53)
NPV	0.97 (0.97–0.98)	0.98 (0.98–0.98)	0.98 (0.98–0.98)	0.98 (0.98–0.98)	0.97 (0.96–0.97)	0.98 (0.97–0.98)	0.99 (0.98–0.99)	0.97 (0.97–0.98)	0.98 (0.97–0.98)	0.98 (0.98–0.98)
Youden’s index^2^	0.68 (0.66–0.7)	0.75 (0.73–0.77)	0.74 (0.70–0.77)	0.72 (0.71–0.73)	0.66 (0.61–0.70)	0.71 (0.70–0.72)	0.77 (0.67–0.84)	0.71 (0.69–0.73)	0.72 (0.70–0.74)	0.73 (0.71–0.75)

COPD: chronic obstructive pulmonary disease. NPV: negative predictive value. PPV: positive predictive value

^1^ Asthma, COPD, diabetes, eczema, hypertension, rhinitis, sleep disorder

^2^ Sensitivity + specificity—1

**Table 5:** Estimates and 95% confidence interval (95% CI) of sensitivity, specificity, positive predictive value, negative predictive value, and Youden’s index by groups of comorbidity for clinician-diagnosed COVID-19 against real-time reverse transcriptase-polymerase chain reaction (RT-PCR) diagnosis of COVID-19

	“Severe” comorbidities^1^			“Moderately severe” comorbidities^2^			“Mild” comorbidities^3^		
	0 *n* = 39,262 Estimate (95% CI)	1 *n* = 3164 Estimate (95% CI)	2 *n* = 195 Estimate (95% CI)	0 *n* = 31,093 Estimate (95% CI)	1 *n* = 10,450 Estimate (95% CI)	2 *n* = 1078 Estimate (95% CI)	0 *n* = 21,790 Estimate (95% CI)	1 *n* = 13,818 Estimate (95% CI)	≥ 2 *n* = 7013 Estimate (95% CI)
Sensitivity	0.78 (0.77–0.79)	0.83 (0.78–0.88)	0.69 (0.44–0.86)	0.78 (0.76–0.79)	0.80 (0.77–0.83)	0.84 (0.73–0.91)	0.78 (0.76–0.80)	0.79 (0.77–0.81)	0.78 (0.75–0.81)
Specificity	0.93 (0.93–0.93)	0.93 (0.92–0.94)	0.94 (0.90–0.97)	0.93 (0.93–0.93)	0.93 (0.93–0.94)	0.93 (0.91–0.94)	0.94 (0.94–0.94)	0.93 (0.93–0.94)	0.91 (0.91–0.92)
PPV	0.54 (0.53–0.56)	0.47 (0.42–0.52)	0.52 (0.32–0.72)	0.55 (0.54–0.57)	0.50 (0.48–0.53)	0.41 (0.33–0.50)	0.56 (0.54–0.57)	0.54 (0.52–0.57)	0.49 (0.46–0.52)
NPV	0.98 (0.97–0.98)	0.99 (0.98–0.99)	0.97 (0.93–0.99)	0.97 (0.97–0.98)	0.98 (0.98–0.99)	0.99 (0.98–0.99)	0.98 (0.98–0.98)	0.98 (0.97–0.98)	0.97 (0.97–0.98)
Youden’s index^4^	0.71 (0.70–0.72)	0.76 (0.70–0.81)	0.63 (0.39–0.81)	0.71 (0.69–0.72)	0.73 (0.70–0.76)	0.77 (0.66–0.84)	0.72 (0.70–0.74)	0.72 (0.70–0.74)	0.69 (0.66–0.72)

NPV: negative predictive value. PPV: positive predictive value

^1^ Chronic obstructive pulmonary disease (COPD) or diabetes

^2^ Asthma or hypertension

^3^ Eczema, rhinitis, or sleep disorder

^4^ Sensitivity + specificity – 1

When we excluded patients who got clinician diagnosis prior to when RT-PCR diagnosis became commonly used in Sweden (28th October 2020), the above results, were overall comparable (Additional file 1: Tables S1–S3).

## Discussion

### Summary of key findings

By comparing clinician diagnosis of COVID-19 based on the use of recommended ICD-10 codes with RT-PCR diagnosis, the results from the current study indicate that clinicians were able to correctly classify 78% of true COVID-19 cases (identified by RT-PCR), while 93% of those ruled out by RT-PCR were correctly classified as negative by clinicians. Furthermore, while comparison of clinician diagnosis with RT-PCR did not differ between males and females, there were differences by age, and pre-COVID BMI, COPD, asthma, and presence of comorbidities.

### Strengths and limitations of the study

The present study has several strengths. The sample size was relatively large and representative of the general adult population of western Sweden. RT-PCR and clinician diagnosis data were gathered from databases with comprehensive coverage. Furthermore, we included several important background factors that have previously been suggested [1–3] to affect the risk of contracting COVID-19. However, some limitations should be considered. The pre-COVID-19 data were collected at least 4 years prior to COVID-19 infection. It is possible that comorbidity, weight, and smoking status have changed during this period. Furthermore, data on weight, height, and comorbidities were assessed using a self-administered questionnaire, a data source which can be prone to misinterpretation [4] and inaccurate assessments by respondents. Several potentially important background factors, such as vaccination status, were unavailable and thus not included in the current analyses. The COVID-19 diagnosis data for this study covered the time up until the end of September 2021. Later data, especially for the large but less clinically severe omicron waves in 2022, could have potentially added valuable data to assess how clinician diagnosis has changed as the disease has changed. On the other hand, full coverage testing for COVID-19 in Sweden during the omicron outbreak has diminished. Finally, it is unclear to what extent clinician diagnosis was influenced by the physician knowing the result of the RT-PCR test prior to the clinical diagnosis. We assume that in many cases, the positive RT-PCR result was available to the physician (and especially if specifically ordered by the doctor), but there may be situations where clinical diagnosis was set first and RT-PCR result was obtained only later. In addition, some patients, generally with mild symptoms, might have not had appointment when having positive RT-PCR result. These may be considered as potential biases to our study. In Sweden, RT-PCR test is the most common test offered, but there are also antigen tests in a small scale, much of these being self-tests. It is unclear as to what extent the antigen test results were available to the physician.

### Comparison of findings with previous studies

The use of RT-PCR and a nasopharyngeal swab is the gold standard for detecting SARS-COV-2 infection, but there have been few studies investigating the accuracy of COVID-19 diagnosis, the majority of which compared the accuracy of computed topography (CT) with RT-PCR [16–18].

In the United States, Blatz et al. [19], conducted a validation study among paediatric inpatients and revealed that clinician diagnosis through the ICD code of U07.1 had an 89.7% sensitivity for identifying those with RT-PCR confirmed COVID-19 infection, as well as specificity of 99.9%, PPV of 95.5%, and NPV of 99.7% [19]. The sensitivity of 78% obtained in our study in Sweden is lower than the value obtained by Blatz et al. [19], whereas specificity of clinician diagnosis was similarly high in both countries. The difference in sensitivity values found in our study and the study conducted in the United States could potentially be attributed to differences in the study population. While our findings were based on a population-based sample of randomly selected adults and clinician diagnosis of COVID-19 from both primary and secondary care, the study by Blatz et al. [19] on the other hand was a single-centre study of children recruited from inpatient department, thus was not population focused. Moreover, why Blatz and colleagues used only one of the recommended ICD codes (U07.1) to defined clinician diagnosis of COVID-19, the ICD codes used in our study were more comprehensive, including U07.1, U07.2, U08.9, U09.9, and U10.9.

With our data, the PPV of clinician diagnosis of COVID-19 was estimated at 54% (95% CI 53–55). This estimate was substantially lower than that of the study by Bodilsen et al. [20] in which medical records of 710 patients (median age of 61 years) admitted to departments of infectious diseases in Danish hospital from 27 February to 4 May 2020 with an ICD-10 diagnosis code of COVID-19 were reviewed. They found an overall PPV of 99% (95% CI 99–100) for clinician diagnosis. This remained consistently high across all subgroups, including gender, age groups, calendar period, and when stratified by diagnosis code and department [20]. Since the predictive values of a test or diagnostic tool are subject to variation in the prevalence of the disease in the population, this could explain the differences in the PPV found in the Danish study and that found in our study. The Danish study was conducted among mainly older adults (mean age of 61 years) at a time when the COVID-19 pandemic was at its peak in Denmark. The population of WSAS was more encompassing, including adults from at least 20 years and upward, most subjects within 30–60 years of age. According to some studies, older adults suffer disproportionately from the most severe outcomes of COVID-19 [21]. With age comes additional pre-existing conditions, making older adults more vulnerable to developing a severe form of COVID-19 infection and possibly more predisposed to the infection among them [21].

### Interpretation of findings

Our findings indicate that clinician diagnosis of COVID-19 in a general adult population is adequate. Given the high cost, slow test turn-around, and varying test capacity, diagnosis by a clinician’s assessment can be a useful supplement at the population level. While the accuracy of clinician diagnosis did not differ between males and females, age-related differences were observed, particularly between the youngest and oldest old groups. This age difference could be due to older patients having a higher probability of presenting to the hospital following a COVID-19 infection than young adults. In a systematic review by Israfil et al. [22], older COVID-19 patients had faster disease progression, higher risk of severe heart attack, higher ICU admission rate, and higher mortality rate than in younger patients; factors that could drive more frequent clinical contacts than among younger adults. In resource-constrained settings where RT-PCR test kits are limited, our findings are reassuring: clinicians are able to diagnose older patients over the age of 60 years to complement for unavailability of RT-PCR tests given the high sensitivity in this age group.

Sensitivity and PPV were higher in those without than in those with asthma, but the specificity and NPV were similar. In contrast, the sensitivity of clinician diagnosis was higher in those with COPD than in those without COPD, while the PPV was higher in those without COPD than in those with COPD, the specificity and NPV being similar. It is unclear the reasons for the contrasting findings between patients with asthma and those with COPD, but studies have shown that adults with COPD are more affected by COVID-19 than adults with asthma. Karlson et al. [23] found that severe COVID-19 and mortality were more common among patients with COPD than those with asthma. The larger proportion of adverse outcomes in COPD patients than asthma patients was attributed to the fact that COPD patients had a higher average age than asthma patients. Additionally, COPD patients are more prone to respiratory infections due to reduced innate and adaptive immune responses than asthma patients [24], which would increase the frequency of clinical contacts among COPD patients compared to asthma patients.

While the specificity remained similar, the sensitivity of clinician diagnosis of COVID-19 increased with increasing number of “severe” comorbidities, but not “moderately severe” or “mild” comorbidities. This is not surprising given that underlying health conditions or comorbidities, like hypertension or diabetes mellitus, have been identified as risk factors for COVID-19 and can facilitate a severe course and rapid progression of the disease [25]. This could explain why clinicians are more likely to diagnose COVID-19 in patients with increased number of comorbidities, particularly those with severe comorbidities. These results indicate that clinician diagnosis of COVID-19 is adequate in patients with underlying health conditions or comorbidities, and thus can be used for screening purposes to supplement RT-PCR in this group of patients.

### Clinical implications of findings

The findings of this study indicate that in the general population, clinician diagnosis is adequate and valid for identifying adults with COVID-19, particularly in aged patients and those with underlying health conditions or comorbidities. These results are important and assuring, particularly in areas where RT-PCR for COVID-19 testing is costly or access is insufficient, such as in low-resource settings. There have been reports of a lack of large-scale COVID-19 testing in many Sub-Saharan African countries, as well as long wait times for RT-PCR tests and long turnaround times due to the high volume of requests, frequent stockouts of reagent and sample collection kits, and power outages [26, 27]. In such cases, clinician diagnosis of COVID-19 can supplement RT-PCR for COVID-19 diagnosis, allowing for timely provision of appropriate treatment as well as advice on prevention and isolation strategies to inform disease control response. However, this should be used with caution and should not entirely replace RT-PCR for COVID-19 diagnosis seeing as some of the symptoms of COVID-19 overlap with those of common infections, such malaria, common cold, dengue, and pneumonia, making diagnosis difficult without an appropriate diagnostic test [26]. Furthermore, given that our produced accuracy estimates are based on population-level data, caution should be taken in interpreting the data in the clinical setting, for which clinical studies are imperative. Overall, the estimates of the Youden’s index in the total population and by the examined subgroups were generally above 70%, well above the benchmark of 50% for an acceptable diagnostic test. This means that clinician diagnosis of COVID-19 using the recommended ICD codes has an acceptable balance between specificity and sensitivity.

## Conclusion

The accuracy of clinician’s diagnosis for COVID-19 is adequate at the population level for adults, regardless of gender, pre-COVID-19 BMI, and obstructive airway diseases, thus can be used for screening purposes to supplement RT-PCR, particularly among aged adults and those with increased number of comorbidities. Pre-COVID-19 factors may influence COVID-19 diagnosis based on diagnostic method. Such information can be useful for planning future research and screening efforts for COVID or other similar outbreaks.

## Supplementary Information

**Additional file 1.** Results from sensitivity analyses.

## Data Availability

The datasets generated and/or analysed during the current study are not publicly available due participants consent not allowing public availability of the data, but are available from the corresponding author on reasonable request.
