# Supplementary material for: Comparison of clinician diagnosis of COVID-19 with real time polymerase chain reaction in an adult-representative population in Sweden
Source: Respir Res. 2023 Jan 11;24:10. doi: 10.1186/s12931-023-02315-7 (PMC9832414; doi:10.1186/s12931-023-02315-7)
Supplement: Supplementary file 1 — Additional file 1. Results from sensitivity analyses. [file 12931_2023_2315_MOESM1_ESM.docx]

#### **eTable 1. Estimates and 95% conﬁdence interval (CI) of sensitivity, speciﬁcity, positive predictive value (PPV), negative predictive value (NPV) and Youden’s index in all patients, gender and age at diagnosis for COVID-19 clinical diagnosis against PCR, in a subsample where physician-diagnosed COVID-19 patients with diagnosis prior to the time at which PCR diagnosis became as common as physician diagnosis were removed.**

|  | **All**  *N*=41,167  Estimate (95% CI) | **Gender** | | **Age groups** | | | |
| --- | --- | --- | --- | --- | --- | --- | --- |
|  |  | **Males**  *n*=18,830  Estimate (95% CI) | **Females**  *n*=22,337  Estimate (95% CI) | **≤30 years**  *n*=2,632  Estimate (95% CI) | **31-44 years**  *n*=8,483  Estimate (95% CI) | **45-60 years**  *n*=11,639  Estimate (95% CI) | **>60 years**  *n*=18,413  Estimate (95% CI) |
| **Sensitivity** | 0.77 (0.75-0.78) | 0.77 (0.75-0.79) | 0.76 (0.74-0.78) | 0.67 (0.61-0.72) | 0.71 (0.68-0.74) | 0.79 (0.77-0.81) | 0.83 (0.81-0.86) |
| **Specificity** | 0.96 (0.96-0.96) | 0.97 (0.96-0.97) | 0.96 (0.95-0.96) | 0.94 (0.93-0.95) | 0.95 (0.94-0.95) | 0.95 (0.94-0.95) | 0.98 (0.97-0.98) |
| **PPV** | 0.66 (0.64-0.67) | 0.69 (0.67-0.71) | 0.63 (0.61-0.65) | 0.63 (0.58-0.68) | 0.66 (0.63-0.69) | 0.68 (0.66-0.71) | 0.62 (0.59-0.65) |
| **NPV** | 0.98 (0.98-0.98) | 0.98 (0.98-0.98) | 0.98 (0.97-0.98) | 0.95 (0.94-0.96) | 0.96 (0.95-0.96) | 0.97 (0.97-0.97) | 0.99 (0.99-0.99) |
| **Youden’s index^1^** | 0.73 (0.72-0.74) | 0.74 (0.72-0.76) | 0.72 (0.7-0.74) | 0.61 (0.56-0.66) | 0.66 (0.63-0.69) | 0.74 (0.72-0.76) | 0.81 (0.78-0.83) |

^1^ Youden’s index (sensitivity + speciﬁcity – 1).

The 95% conﬁdence intervals (CI) of the estimates were computed using Wilson’s method.

#### **eTable 2. Estimates and 95% conﬁdence interval (CI) of sensitivity, speciﬁcity, positive predictive value (PPV), negative predictive value (NPV) and Youden’s index by pre-COVID BMI, comorbidity, and asthma/COPD, in a subsample where physician-diagnosed COVID-19 patients with diagnosis prior to the time at which PCR diagnosis became as common as physician diagnosis were removed.**

|  | **Body mass index (BMI), kg/m^2^** | | | **Number of co-morbidity conditions** | | | **Asthma (physician-diagnosed)** | | **COPD (physician-diagnosed)** | |
| --- | --- | --- | --- | --- | --- | --- | --- | --- | --- | --- |
|  | **<25**  *n*=20,409  Estimate (95% CI) | **25-29.99…**  *n*=15,142  Estimate  (95% CI) | **≥30**  *n*=5,616  Estimate  (95% CI) | **0**  *n*=16,416  Estimate  (95% CI) | **1**  *n*=12,018  Estimate  (95% CI) | **≥ 2**  *n*=12,733  Estimate  (95% CI) | **No**  *n*=37,688  Estimate  (95% CI) | **Yes**  *n*=3,479  Estimate  (95% CI) | **No**  *n*=39,965  Estimate  (95% CI) | **Yes**  *n*=1,202  Estimate  (95% CI) |
| **Sensitivity** | 0.73 (0.71-0.75) | 0.8 (0.78-0.82) | 0.79 (0.75-0.82) | 0.75 (0.73-0.77) | 0.77 (0.75-0.8) | 0.78 (0.75-0.8) | 0.77 (0.76-0.78) | 0.72 (0.67-0.77) | 0.76 (0.75-0.78) | 0.81 (0.7-0.89) |
| **Specificity** | 0.96 (0.96-0.96) | 0.96 (0.96-0.96) | 0.96 (0.95-0.97) | 0.96 (0.96-0.97) | 0.96 (0.96-0.96) | 0.96 (0.95-0.96) | 0.96 (0.96-0.96) | 0.95 (0.94-0.96) | 0.96 (0.96-0.96) | 0.96 (0.94-0.97) |
| **PPV** | 0.65 (0.63-0.67) | 0.66 (0.64-0.68) | 0.65 (0.61-0.69) | 0.68 (0.66-0.7) | 0.66 (0.63-0.69) | 0.62 (0.6-0.65) | 0.66 (0.64-0.67) | 0.62 (0.57-0.67) | 0.66 (0.65-0.67) | 0.5 (0.4-0.6) |
| **NPV** | 0.97 (0.97-0.98) | 0.98 (0.98-0.98) | 0.98 (0.98-0.98) | 0.97 (0.97-0.98) | 0.98 (0.97-0.98) | 0.98 (0.98-0.98) | 0.98 (0.98-0.98) | 0.97 (0.96-0.97) | 0.98 (0.97-0.98) | 0.99 (0.98-0.99) |
| **Youden’s index^1^** | 0.69 (0.67-0.71) | 0.76 (0.74-0.78) | 0.75 (0.71-0.78) | 0.71 (0.69-0.73) | 0.73 (0.7-0.75) | 0.74 (0.71-0.76) | 0.73 (0.72-0.74) | 0.67 (0.62-0.72) | 0.72 (0.71-0.73) | 0.77 (0.66-0.85) |

^1^ Youden’s index (sensitivity + speciﬁcity - 1).

The 95% conﬁdence intervals (CI) of the estimates were computed using Wilson’s method.

#### **eTable 3. Estimates and 95% conﬁdence interval (CI; computed using Wilson’s method) of sensitivity, speciﬁcity, positive predictive value (PPV), negative predictive value (NPV) and Youden’s index by groups of comorbidity, in a subsample where physician-diagnosed COVID-19 patients with diagnosis prior to the time at which PCR diagnosis became as common as physician diagnosis were removed.**

|  | **"Severe" comorbidities (COPD, diabetes)** | | | **"Moderately severe" comorbidities (asthma, hypertension)** | | | **"Mild" comorbidities (eczema, rhinitis, sleep disorder)** | | |
| --- | --- | --- | --- | --- | --- | --- | --- | --- | --- |
|  | **0**  *n*=37,922  Estimate  (95% CI) | **1**  *n*=3,056  Estimate  (95% CI) | **≥ 2**  *n*=189  Estimate  (95% CI) | **0**  *n*=30,023  Estimate  (95% CI) | **1**  *n*=10,106  Estimate  (95% CI) | **≥ 2**  *n*=1,038  Estimate  (95% CI) | **0**  *n*=21,123  Estimate  (95% CI) | **1**  *n*=13,339  Estimate  (95% CI) | **≥ 2**  *n*=6,705  Estimate  (95% CI) |
| **Sensitivity** | 0.76 (0.75-0.78) | 0.81 (0.75-0.86) | 0.67 (0.42-0.85) | 0.76 (0.74-0.77) | 0.79 (0.76-0.82) | 0.82 (0.7-0.9) | 0.76 (0.74-0.78) | 0.77 (0.75-0.79) | 0.76 (0.73-0.79) |
| **Specificity** | 0.96 (0.96-0.96) | 0.96 (0.95-0.97) | 0.97 (0.93-0.99) | 0.96 (0.96-0.96) | 0.96 (0.96-0.97) | 0.96 (0.94-0.97) | 0.96 (0.96-0.97) | 0.96 (0.96-0.96) | 0.95 (0.95-0.96) |
| **PPV** | 0.66 (0.65-0.68) | 0.57 (0.51-0.62) | 0.67 (0.42-0.85) | 0.67 (0.65-0.68) | 0.63 (0.6-0.66) | 0.53 (0.42-0.63) | 0.67 (0.65-0.69) | 0.66 (0.63-0.68) | 0.62 (0.58-0.65) |
| **NPV** | 0.98 (0.97-0.98) | 0.99 (0.98-0.99) | 0.97 (0.93-0.99) | 0.97 (0.97-0.98) | 0.98 (0.98-0.99) | 0.99 (0.98-0.99) | 0.98 (0.98-0.98) | 0.98 (0.97-0.98) | 0.97 (0.97-0.98) |
| **Youden’s index^1^** | 0.72 (0.71-0.73) | 0.77 (0.71-0.82) | 0.64 (0.39-0.83) | 0.72 (0.7-0.74) | 0.75 (0.72-0.78) | 0.78 (0.66-0.86) | 0.72 (0.7-0.74) | 0.73 (0.71-0.75) | 0.71 (0.67-0.74) |

^1^ Youden’s index (sensitivity + specificity – 1).
